# PROstate Multicentre External beam radioTHErapy Using a Stereotactic boost: the PROMETHEUS study protocol

**DOI:** 10.1186/s12885-018-4511-6

**Published:** 2018-05-24

**Authors:** Matthew Richardson, Mark Sidhom, Sarah Gallagher, Mel Grand, David Pryor, Joseph Bucci, Lee Wilton, Sankar Arumugam, Sarah Keats, Jarad M. Martin

**Affiliations:** 1 Calvary Mater Newcastle, Newcastle, NSW Australia; 2 Liverpool and Macarthur Cancer Therapy Centres, Sydney, NSW Australia; 3 Ingham Institute, Liverpool, NSW Australia; 4 Princess Alexandra Hospital, Brisbane, QLD Australia; 5 St. George Hospital Cancer Centre, Sydney, NSW Australia

**Keywords:** Radiation oncology, Prostatic neoplasms, Dose Hypofractionation, Radiosurgery, Planning techniques

## Abstract

**Background:**

High Dose Rate Brachytherapy (HDRB) boost is a well-established treatment for prostate cancer (PC). We describe the PROstate Multicentre External beam radioTHErapy Using Stereotactic boost (PROMETHEUS) study. Non-surgical stereotactic techniques are used to deliver similar doses to HDRB boost regimens with a dose escalation sub-study.

**Methods:**

Eligible patients have intermediate or high risk PC. PROMETHEUS explores the safety, efficacy and feasibility of multiple Australian centres cooperating in the delivery of Prostate Stereotactic Body Radiotherapy (SBRT) technology. A SBRT boost component Target Dose (TD) of 19Gy in two fractions is to be delivered, followed by a subsequent EBRT component of 46Gy in 23 fractions. Once accrual triggers have been met, SBRT doses can be escalated in 1 Gy increments to a maximum of 22Gy in two fractions. Patient safety will also be measured with the rate of both acute and late moderate to severe Gastro-Intestinal (GI) and Genito-Urinary (GU) Common Terminology Criteria for Adverse Events (CTCAE) toxicities as well as patient reported quality of life. Efficacy will be assessed via biochemical control after 3 years.

**Discussion:**

PROMETHEUS aims to generate evidence for a non-surgical possible future alternative to HDRB boost regimens, and introduce advanced radiotherapy techniques across multiple Australian cancer centres.

**Trial registration:**

The study was retrospectively registered on the ANZCTR (Australian New Zealand Clinical Trials Registry) with trial ID: ACTRN12615000223538.

## Background

Prostate Cancer (PC) is a common malignancy in Australian men. In men with localized disease, external beam radiotherapy (EBRT) is a regularly used management option.

Conventional fraction sizes of 1.8–2 Gy per day are considered to be a standard treatment approach. There is, however, a growing body of evidence to support the efficacy and safety of larger doses per fraction [1–3]. This stems from clinical radiobiological data suggesting that PC has a low alpha/beta ratio (ABR), and as such a therapeutic ratio could be exploited between the differential fraction size sensitivity of the prostate and the adjacent critical structures, especially the rectum [4, 5].

A fundamental concept in radiation oncology is that Tumour Control Probability (TCP) increases as a function of radiation dose. This principle has been validated in several randomized trials of EBRT, with all studies demonstrating an improvement in Prostate Specific Antigen (PSA) control Biological Non-Evidence of disease (bNED) [6, 7]. However, this increased efficacy came at the cost of increased late rectal toxicity [8], and has spurred the development of new technologies to escalate dose to the prostate, while sparing neighbouring critical structures.

Brachytherapy has a history spanning over two decades as one method of escalating dose to the prostate. Being surgically implanted into the prostate, brachytherapy is an extremely conformal treatment approach, allowing radiotherapy dose to be tailored very precisely to the required volume. Either Low Dose Rate Brachytherapy (LDRB) or High Dose Rate Brachytherapy (HDRB) can also be combined with 45–50 Gy of conventionally fractionated EBRT as a dose escalation strategy. Data has been published from several Australian centres confirming the feasibility, efficacy, and low rectal toxicity associated with a HDRB approach [9, 10].

Despite this evidence, brachytherapy dose escalation is only available in a relatively limited number of cancer hospitals in Australia. This is mainly due to the need for specialised personnel, theatre access, concerns regarding urethral stricture rates and the high cost of maintaining a HDRB unit [9].

All radiotherapy centres in Australia have the capacity to deliver escalated doses of radiotherapy of 76 Gy or higher. To do this safely, newer technologies have been widely introduced over the last decade. Three Dimensional (3D) planning techniques permit more detailed anatomical information to be incorporated into the design of a patient’s treatment. Image Guided Radiotherapy (IGRT) allows more precise delivery of radiation dose to the prostate [11]. Intensity Modulated Radiotherapy (IMRT) using dynamic beam shaping and inverse planning approaches facilitates shaping the higher doses of radiation to irregular shapes [12]. The combination of all of these techniques has led to reduced rates of late grade 2–3 rectal toxicity in the dose escalated setting from 26% reported in the most mature of the randomized studies [6] to ~ 6% in single institution reports [13]. As a result, these approaches feature in current Australasian guidelines and are now widely practiced in Australia [4].

Despite recent advances, it is likely that we are reaching a threshold effect with EBRT alone.

The highest reported conventional doses delivered are 86.4 Gy in 1.8 Gy fractions from MSKCC [14]. Retrospective comparison of this regimen with brachytherapy boost alternatives showed markedly inferior biochemical and metastatic disease control [15]. Advances in EBRT including IMRT and IGRT, as well as emerging evidence for hypofractionation in PC have opened up an avenue to explore Stereotactic Body Radiotherapy (SBRT) as an alternative to HDRB as a prostate radiation dose escalation strategy.

## Methods/Design

**Fig. 1:**
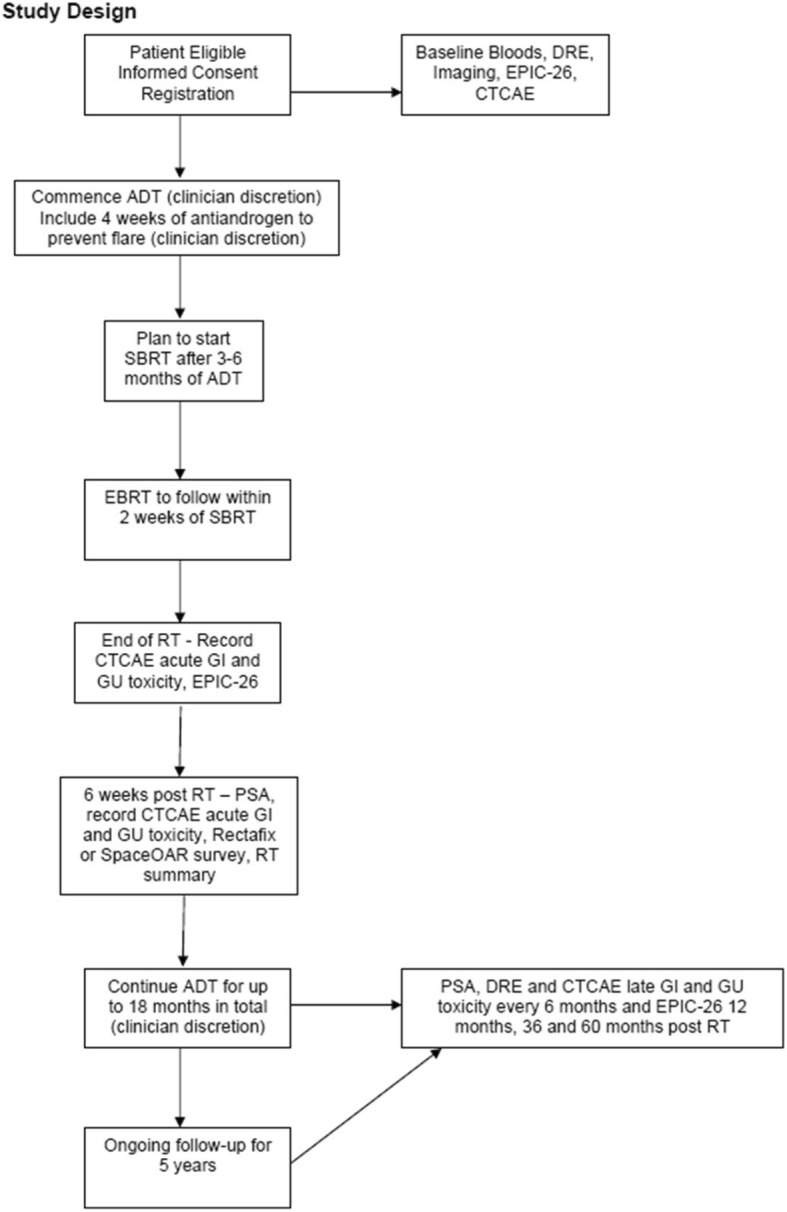
PROMETHEUS trial study design

PROMETHEUS is a Phase 2 multicentre clinical trial exploring a stereotactic Radiotherapy Boost to the prostate with fractionated external beam radiotherapy. We aim to test the following hypotheses; (1) That radiotherapy dose escalation to the prostate via a SBRT boost is safe using a linear accelerator in the multi-centre setting. (2) That radiotherapy dose escalation to the prostate via a SBRT boost can be increased in a stepwise manner and finally, (3) that radiotherapy dose escalation to the prostate via a SBRT boost is feasible using a Linear Accelerator in the multi-centre setting. The study design is shown in Fig. 1.

### Key selection criteria

#### Inclusion criteria

Men capable of giving informed consent with a histological diagnosis of intermediate or high risk prostate adenocarcinoma as defined by any one of: a) Baseline PSA 10–20, Gleason grade 7 disease, Clinical stage T2b-c OR b) Baseline PSA ≥20 Gleason grade 8–10 disease, Clinical stage T3. Once deemed eligible, recommended 6 months Androgen Deprivation Therapy (ADT) for unfavourable intermediate risk or low-high risk men (1 high risk factor), and 18–24 months for men with multiple high risk factors.

#### Exclusion criteria

Patients having received previous pelvis radiotherapy, ECOG performance status > 1, hip prosthesis in-situ, inability to have a Magnetic Resonance Imaging (MRI), clinical stage T4 disease, presence of inflammatory bowel disease or severe obstructive urinary symptoms, and finally an inability to meet planning objectives.

### Objectives

The primary objectives of the study are to determine the safety and efficacy of a SBRT prostate boost. Safety will be established if the cumulative rate of either acute or late GI or GU toxicity is equivalent to or less than previously reported in the HDRB or dose escalated IGRT EBRT literatures. Efficacy will be assessed via biochemical control after 3 years.

The secondary objective of the study is to ensure that the rates of grade 3 or higher acute CTCAE GI or GU toxicity does not increase with increasing dose delivery to the Clinical Target Volume (CTV).

Multicentre feasibility will be demonstrated if 3 or more centres contribute 5 or more patients to the study.

### Treatment planning

A Rectal Displacement Device (RDD) is to be used with the main options being the transperineal application of SpaceOAR, or transanal insertion of a Rectafix™ to increase distance from rectum to prostate [16, 17].

### Simulation

Intraprostatic fiducial markers will be inserted at least 7 days prior to treatment planning. An appropriate RDD is selected for rectal separation. A temporary In-Dwelling urinary Catheter (IDC) can be inserted for planning scans to aid urethral contouring at clinician’s discretion. Patients will be instructed to follow bladder and bowel preparation to achieve an empty rectum and full bladder.

A planning Computed Tomography (CT) scan is acquired in both SBRT and EBRT treatment positions. Minimum CT slice thickness of 2.5 mm, scanning from L4 to include whole pelvis to below the perineum. A SBRT planning MRI with RDD in-situ is to be performed within 1 h of CT to minimize effect of prostate deformation caused by variable bowel filling.

### SBRT treatment planning

CT-MRI fusion of planning scans using prostatic fiducial registration is performed.

The CTVsbrt is the prostate plus any observed extraprostatic disease, either due to T3a extracapsular extension (ECE) or seminal vesicle invasion (SVI). For SVI include disease observed on MRI only for the boost. The SBRT Planning Target Volume (PTVsbrt) expansion is 5 mm in all directions from CTVsbrt, except posteriorly where it is 3 mm.

Table 1 lists the critical structure contouring guidelines.

**Table 1:** Critical structure contouring guidelines

Structure name	Description
Rectal Wall	Contour as a 3 mm thick wall structure from recto-sigmoid junction to lower aspect of ischial tuberosities.
Rectal Mucosa	Solid structure corresponding to the internal cylindrical space central to the inner surface of the rectal wall.
Rectum Posterior Wall	This is the most posterior 15 mm of rectal wall
Bladder	Contour the whole organ as a solid structure.
Penile Bulb	Contour from MRI.
Prostatic urethra Planning Target at Risk Volume (PRV)	Contour urinary IDC within prostate, and add 1 mm radial expansion for the PRV. If no IDC used, estimate urethral position, and add 3 mm radial expansion.
Neck of Femur	Contour the Left and Right NOF as solid structures to the level of the ischial tuberosity.

Inverse planned IMRT or related modulated dose techniques (Tomotherapy, Cyberknife [CK] or Volumetric Modulated Arc Radiotherapy [VMAT]) are required. For static field IMRT, between 5 and 8 fields recommended. Photon energies of 6–10 MV, with higher energies not recommended due to the higher potential for neutron scatter with the large daily doses. An exception is patients with large separations where 1–2 higher energy beams may be helpful in improving dose distribution, but only if all other beams are 6–10 MV. Non-coplanar beams are allowed. VMAT may provide the advantage of more rapid treatment time reducing the need for interim imaging to manage intrafraction motion.

SBRT dose constraints should be met as per Table 2:

**Table 2:** SBRT dose constraints. (TD = Target dose)

Constraint	Per-Protocol	Minor Variation	Major Variation
CTVsbrt D98^a^	> 100% TD	95–100% TD	< 95% TD
PTVsbrt D50^a^	< 105% TD	105–110% TD	> 110% TD
PTVsbrt D90^a^	> 100% TD	95–100% TD	< 95% TD
PTVsbrt D95^a^	> 95% TD	90–95% TD	< 90% TD
PTVsbrt D99^a^	> 16 Gy	15–16 Gy	< 15 Gy
PTVsbrt Dmax to 0.1cc	< 110% TD	110–120% TD	> 120% TD
PTVsbrt Dmax	Not within a critical structure		
Rectal Wall Dmax to 0.1cc	< 17 Gy	17–17.5 Gy	> 17.5 Gy
Rectal Wall V16 Gy	< 0.5cc	0.5-1cc	> 1cc
Rectal Wall V14 Gy	< 3cc	3-5 cc	> 5cc
Rectal Wall V12 Gy	< 30%	30–40%	> 40%
Rectal Wall V10 Gy	< 40%	40–50%	> 50%
Rectal Wall V8 Gy	< 60%	60–70%	> 70%
Rectal Mucosa Dmax to 0.1cc	< 15 Gy	15–15.5 Gy	> 15.5 Gy
Rectal Mucosa V14 Gy	< 0.5cc	0.5-1cc	> 1cc
Rectum Posterior Wall	< 8.5 Gy	8.5–9.5 Gy	> 9.5 Gy
Bladder Dmax to 0.1cc	< 110% TD	110–120% TD	> 120% TD
Bladder V19 Gy	< 10cc	10-15cc	> 15cc
Bladder V17 Gy	< 15%	15–20%	> 20%
Bladder V9 Gy	< 50%	50–60%	> 60%
Urethra PRV Dmax to 0.1cc	< 110% TD	110–115% TD	> 115% TD
Urethra PRV V105% TD	< 5%	5–15%	> 15%
Neck of Femurs Dmax to 0.1cc	< 8 Gy	8–9 Gy	9 Gy
Penile Bulb Dmax to 0.1cc (Recommended)	100% TD	100–105% TD	> 105% TD
Penile Bulb V10 Gy (Recommended)	<3cc	3-5cc	> 5cc
Intermediate Dose Spillage: ratio of volumes receiving 50% TD to 100% TD	< 4	4–5	> 5
Total Monitor Units	< 3× Dose in cGy	3–3.5× Dose in cGy	> 3.5× Dose in cGy
High Dose Conformation: Volume receiving 100% of TD divided by volume of PTVsbrt	< 1.1	1.1–1.2	> 1.2

^a^Where Urethra has been limited to 19 Gy, these volumes may exclude the urethra: eg. CTVsbrt D98 = CTVsbrt – Urethra PRV D98

**Fig. 2:**
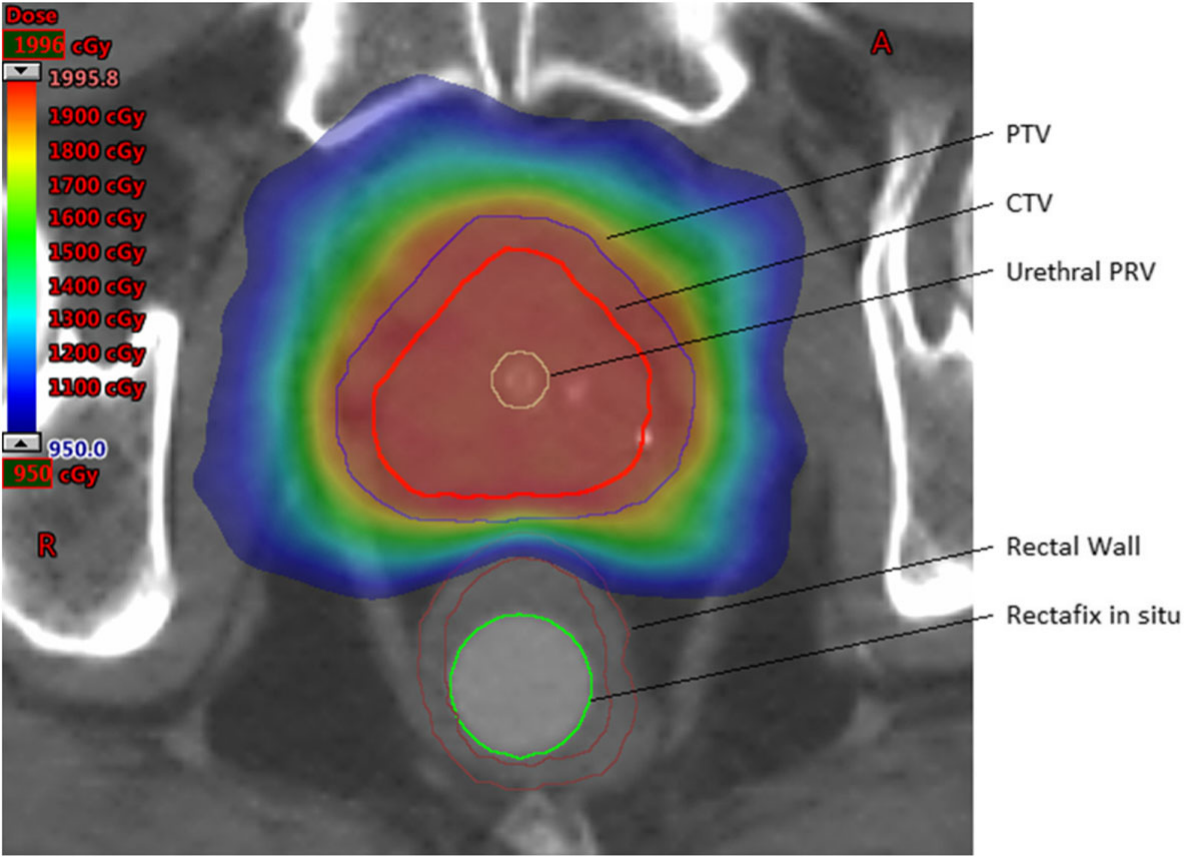
Transverse CT view of example dose distribution adhering to planning constraints with Rectafix™ displacing rectum posteriorly

**Fig. 3:**
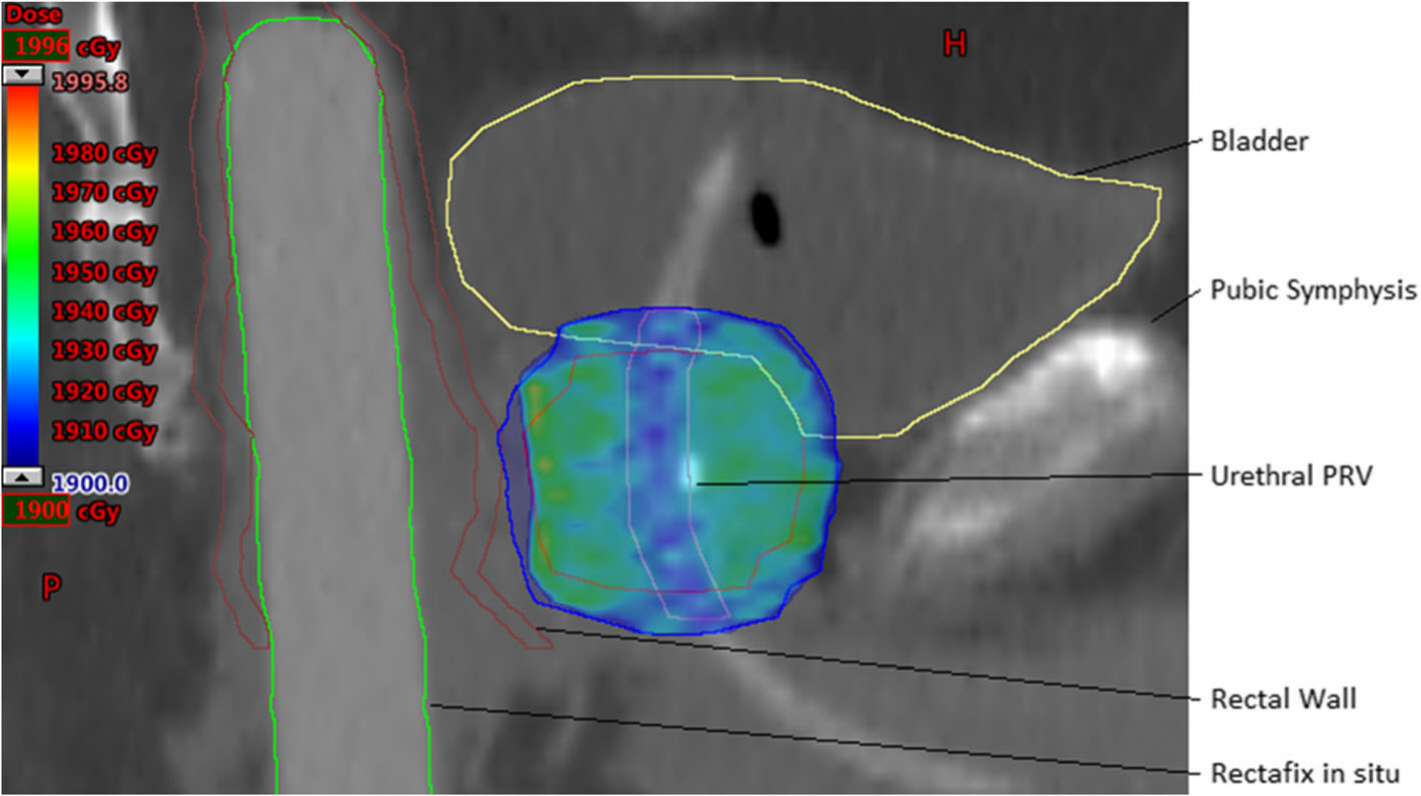
Sagittal CT view of example dose distribution. Note the high dose sparing of the Urethral PRV, and posterior PTVsbrt dose compromise to meet rectal dose constraints

Aim to encompass the entire PTVsbrt with 19Gy minimum. However, PTVsbrt coverage may be compromised posteriorly to a minimum of 16Gy only where necessary in order to meet rectal dose constraints. Aim for 16Gy isodose line to only encompass posterior PTVsbrt where there is overlap between PTVsbrt and Rectum (see Figs. 2 and 3).

The mechanism for the stepwise SBRT dose escalation is as follows:

A minimum of 20 men in total are to complete treatment without > 15% suffering a grade 3 acute toxicity or any episodes of grade 4 acute toxicity at a particular dose level. The individual centre must also accrue ≥5 patients at the previous dose level prior to exploring dose escalation. Once this accrual trigger has been met, increase the dose by 1 Gy to D98% of the volume CTVsbrt minus urethral PRV. The first dose escalation will therefore be to 20 Gy. This process can be followed a maximum of three times, to a maximum CTVsbrt-Urethral PRV D98 of 22 Gy.

A credentialing “dummy run” will need to be performed on a previous patient data set to demonstrate to an external reviewer the feasibility of achieving these new dose constraints. The first three patients planned at each dose level will be subject to a real-time QA process. An external RO review of target delineation, OAR doses and plan quality shall be undertaken before any planned treatment is delivered.

### EBRT treatment planning

The EBRT component of the treatment package will follow the SBRT. CTVebrt will be at the discretion of the treating clinician. For ECE, a 3 mm margin around the prostate is recommended, excluding the rectal wall. For SVI if no gross T3b disease is detected, the proximal 20 mm of the seminal vesicle should be included. For Lymph-node invasion (LNI), the pelvic nodal RT is recommended if the LNI risk is > 15% as indicated by the MSKCC Prostate Nomogram. If treated, pelvic nodes shall be contoured as per the Radiation Therapy Oncology Group (RTOG) guidelines with a superior limit of the bifurcation of the common iliac arteries, and excluding the pre-sacral nodes as reflected by current surgical recommendations of the extent of an extended lymph node dissection [18]. CTVebrt to PTVebrt expansion will be 5–7 mm, or 7 mm if gross SVI is present around this structure in particular.

46Gy in 23 fractions will be prescribed to 95% of the PTVebrt as per ICRU 83. An IMRT, VMAT or Tomotherapy technique is recommended. Dose constraints are outlined in the accompanying Table 3.

**Table 3:** Phase two EBRT component dose constraints

Structure	Per-Protocol	Minor Variation	Major Variation
PTVebrt D95	> 46 Gy	44–46 Gy	< 44 Gy
Prostatic Urethra PRV	Dmax < 47 Gy	Dmax: 47–50 Gy	Dmax > 50 Gy
Small Bowel	Dmax < 47 Gy	Dmax: 47–50 Gy	Dmax > 50 Gy
Neck of Femur	Dmax < 35 Gy	Dmax: 35–45 Gy	Dmax> 45 Gy
Rectum V45	< 25%	25–35%	> 35%
Bladder V45	< 25%	25–35%	> 35%

### Treatment delivery

Due to potential volume changes during a course of EBRT, as well as evidence of volume changes after HDRB, all patients should receive the boost component of their therapy prior to the fractionated EBRT component [19, 20].

The two SBRT boost fractions are to be separated by a 1 week break. The EBRT component is then to commence 2 weeks after the final boost fraction.

Preparation: Patient to continue same bladder preparation and enema regimen as used during simulation. Use Rectafix™ if utilised during simulation.

IGRT: On-line correction to gold fiducials, prostate and IDC with 0 mm action threshold. All effort should be made to commence treatment as soon as feasible after pre-treatment imaging has been performed.

During SBRT treatment: For techniques where real-time tracking or position monitoring is available (e.g. CK kV or intraprostatic transponders) this should be used. For other approaches, aim to perform repeat orthogonal imaging at least every 4 min, as there is evidence to support that 95% of patients will experience less than 3 mm of intrafraction motion over this timeframe [21]. Since most intrafraction motion will be either in the inferior-superior or anterior-posterior planes, only a lateral Electronic Portal Imaging (EPI) is necessary during treatment [22, 23].

### Data collection

Patients will be assessed at baseline, the completion of treatment, 6 weeks post treatment, and then six monthly thereafter up to 5 years. At all visits, CTCAE GI and GU toxicity will be recorded. PSA will be measured at baseline, and then at all visits following treatment. The main efficacy endpoint is bNED calculated by the Phoenix definition of nadir+ 2 [24]. Any disease relapses or initiation of salvage treatments will also be recorded. Patient related quality of life (EPIC-26), and questionnaires regarding tolerance of treatment will also be collated at baseline, end of treatment, 12 months, 36 months and 60 months.

### Sample size

The actuarial rates of CTCAE late grade 2–3 GI and GU toxicity will be calculated and reported at the 5 year mark. Studies using HDRB boost as well as IG-IMRT suggest rates of 5–15% are achievable. The study will be powered to recruit sufficient numbers to be confident that < 15% of men will have either a grade 2–3 GI or grade 2–3 GU late GU event. The formula is:

$$ \mathrm{n}=\left(1.96/\mathrm{E}\right)2\ \mathrm{p}\left(1\hbox{-} \mathrm{p}\right) $$

Where E is the margin of error. A conservative upper estimate of p here is 0.225 (the probability of an event). E might be 0.05 since that at worst gives a 95% CI as 0.15+/− 0.05. With these figures *n* = 268.

#### Feasibility

The protocol is deemed to be feasible if all of the following criteria are met:

- At least 3 different centres participate.
- Each centre accrues at least 5 patients.
- At least two centres attempt dose escalation.

## Discussion

Several randomized controlled trials have demonstrated superior outcomes of a brachytherapy boost approach for intermediate and high risk PC compared with EBRT alone [25, 26]. Despite this, the use of brachytherapy continues to regress [27]. Concurrently, the wider availability of new technology has created an opportunity to try to replicate brachytherapy boost type radiotherapy treatment plans which can be delivered using standard EBRT equipment. The higher daily doses in the SBRT component are potentially very harmful to normal tissues, hence meticulous attention has been made in this study to minimise the risks to patients. Although three main endpoints are noted, it is the risk of severe toxicity which is prioritized in calculating the necessary patient numbers.

The management of such men is complicated by the integration of ADT, the investigation of newer agents, and the simultaneous investigation of SBRT monotherapy. The PROMETHEUS trial explores whether optimisation of the EBRT component is possible by translating the HDRB boost regimen into a non-invasive equivalent which can be more widely deployed. The structure of a future randomized trial is difficult to predict given the ongoing evolution in practice, however the PROMETHEUS schedule could be flexibly combined with various non-cytotoxic agents given the freedom to do so in the protocol.

## Abbreviations

ABR: Alpha/beta ratio
ADT: Androgen deprivation therapy
AE: Adverse event
ARTG: Australian register of therapeutic goods
bNED: Biological non-evidence of disease
CBCT: Cone beam computerised tomography
CK: Cyberknife
CT: Computerised tomography
CTC AE: Common terminology criteria for adverse events
CTV: Clinical target volume
DRE: Digital rectal examination
EBRT: External beam radiation therapy
ECE: Extracapsular extension
ECOG: Eastern Cooperative Oncology Group
EPI: Electronic portal imaging
GI: Gastro intestinal
GU: Genito urinary
HDRB: High dose rate brachytherapy
ICRU: International commission of radiation units and measurements
IDC: In-dwelling urinary catheter
IGRT: Image guided radiation therapy
IMRT: Image guided radiation therapy
KV: Kilovoltage
LDRB: Low dose rate brachytherapy
MRI: Magnetic resonance imaging
MSKCC: Memorial sloan kettering cancer center
MV: Megavoltage
MVCT: Megavoltage computerised tomography
NOF: Neck of femur
OAR: Organ at risk
PC: Prostate cancer
PCSS: Prostate cancer specific survival
PRV: Planning target at risk volume
PSA: Prostate specific antigen
PTV: Planning target volume
QA: Quality assurance
RDD: Rectal displacement device
RT: Radiation therapy
RTOG: Radiation therapy oncology group
SBRT: Stereotactic body radiotherapy
SVI: Seminal vesicle invasion
VMAT: Volumetric modulated arc therapy
